# Efficiency to enable equitable inclusion in relation to new technology

**DOI:** 10.1590/S1516-31802007000600001

**Published:** 2007-11-01

Evidence-Based Medicine is a process for medical practice in which, starting from a structured question, a search for the best scientific evidence in the literature is conducted and the evidence found is adapted according to the patient, the context and the doctor's experience. In this way, doctors take on the commitment to use the best scientific evidence available for treating their patients. It is important to make it clear that the doctor's experience is fundamental to this, since he will inform the patient about the options that exist, their risks and their benefits. Thus, scientifically informed doctors and patients who have received adequate explanations make decisions together and the moral responsibility for the results is shared. This process benefits both sides: patients, because they feel that they have received sufficient information about the types of treatment that offer the greatest likelihood of being effective; and doctors, because they have gained their patients’ trust and thereby have increased the chances of success for the treatment instituted and prevented unnecessary lawsuits that would end up affecting the country's judicial process itself.

Healthcare decision-making also has economic repercussions, because if the approach that has greatest likelihood of success is recognized, this will avoid waste through approaches that are extremely expensive and do not work. Thus, we need to choose the approach that is most likely to work, and this means that there has to be evidence that it is efficacious, effective, efficient and safe.

An intervention is efficacious if it works under ideal conditions, i.e. within a scenario in which patients follow prescriptions correctly, without unforeseen events such as side effects or lack of money for purchasing the medication. An intervention is effective if it works in the real world, within a scenario in which patients who receive diagnoses, prescriptions, guidance and recommendations that their treatment should be "for the rest of their lives" will leave the consultation with the prescription form, will probably go to compare this medication with others, will check whether they have the money for this and will observe whether there is any adverse effect from using the medication. An intervention is efficient if it is easy to implement and economically viable within the bounds of equity. There is no point in having an intervention that is effective and efficient if it is not safe.

Every year, products potentially capable of costing the Ministry of Health one to two billion dollars a week are launched on the Brazilian market. On the other hand, the treatment to save the life of a tuberculosis patient costs about 100 reais. The demand is always going to be infinite and the resources are always limited. The Brazilian government allocates 20 billion dollars per year to healthcare, while the United States government makes 1.7 trillion dollars per year available.^1^ The fact is that we Brazilian doctors and medical school staff are trained using the academic and decision-making models of the United States, which ignore the costs involved, and we try to practice medicine in the same way as is done in that country. Moreover, patients receive information about the American healthcare system through the media. A conflict is created, involving patients, doctors, attorneys, prosecutors, other lawyers, private healthcare systems and the Ministry of Health itself. This conflict ends up in lawsuits within which it cannot be known whether there is or is not any evidence regarding the effectiveness and safety of the medication.

Lack of knowledge of the concepts described above and about how to assess the evidence creates controversies within a culture in which the decision-making process is based on individual opinions, which ends up resulting in damaging judicial disputes from which society itself comes out the loser.

The solution is to only use efficient interventions and to invest in what is known to work, with scientific evidence that it works. This is indeed the way to achieve efficiency and distribute healthcare benefits to the greatest possible number of people. There could be a revolution in Brazil, without expenditure. It is just a matter of making effective healthcare decisions based on good-quality evidence, improving doctor-patient relationships and improving the education of patients and healthcare professionals. Contrary to what is thought, the solution is not through creating more medical schools, since this is not going to improve patients’ access to healthcare but will only increase the numbers of poorly prepared doctors who have pens in their hands, ready to prescribe any modern interventions. These are usualy, however, expensive and ineffective. It is unacceptable to prescribe a certain drug just because it is more modern. This latter adjective, incidentally, is extremely dangerous in Medicine.

Also within this process, it is important to remember that up to now it is the producer or salesman who is responsible for demonstrating the efficiency and safety of a product, and not the consumer.

Evidence-Based Medicine has a long history. More than 200 years ago, in 1753, James Lind was concerned about the fact that half of the crew-members of British ships were dying of scurvy during their voyages. Therefore, he reviewed the literature and then conducted a clinical trial using the interventions available for treating the disease. He recorded that the sailors who received lime and lemon juice became cured, and this was one of the greatest advances in the history of Medicine. However, it took another 50 years after the publication of this study for the British Empire to start to supply lime and lemon juice to its sailors. This story thus shows that it is not enough to have the science: it needs to be applied in practice, i.e. the evidence needs to be implemented. Otherwise, indemnification lawsuits will arise and patients will fail to be treated using the methods that really work.

A more logical phase of medicine was stimulated in 1910 and was based on physiopathology. Following this logic, patients would need to be treated in accordance with the mechanism for the origin of that disease that was most accepted. This mechanism would be based on epidemiological data, experimental studies in laboratories and doctors’ experience.

However, there are things that are logical but in practice do not work, are expensive and are even harmful. For example, postmenopausal women with the beginnings of atrophy of the ovaries present diminished production of the female hormones progesterone and estrogen, appearance of wrinkles, vaginal dryness and bone fragility. Therefore, if the hormonal deficiency is the cause of everything, hormone replacement should be implemented. All perfectly logical. This logic was followed for three decades and was costing about 500 dollars per patient per year. Some decades on, a controlled study comparing hormone replacement with placebo showed that the group of patients who received hormone replacement presented increased rates of infarct, stroke, invasive breast cancer, thromboembolism and gallstones. There were 30 more cases of these adverse effects per 10,000 women treated with hormone replacement than were found among untreated women. In other words, among the 10 million patients receiving hormones in the United States, there were 30,000 cases with complications due to breast cancer.^2-4^ Once again, the mistake was the lack of randomized controlled clinical trials providing good evidence before making the medication available for clinical practice and on the market. On the other hand, there are also things that are logical, work in clinical practice and are simple and economical, but which are not disseminated. For example, there is good-quality scientific evidence showing that when calcium carbonate, which costs five Brazilian real cents per day, is administered to women during the prenatal period, it prevents preeclampsia, eclampsia and all the complications of hypertension during pregnancy. This approach is followed in the cases of only 12% of pregnant women in São Paulo.^5^ The conclusion is that when there are interests involved, even if the intervention is not effective and safe, the system causes all of society to become aware of it, while when the intervention is effective and economically viable, there is no interest in disseminating this information.

In 1972, Archibald Cochrane received the "mission" of assessing the British healthcare system and he raised large numbers of questions such as: Is amygdalectomy in children efficient and safe? Is cesarean delivery more effective and safer than normal delivery? With such questioning, Archibald Cochrane stirred up Medicine around the world and established the challenge of seeking effectiveness and efficiency. Since then, Medicine has needed to assess the effectiveness and efficiency of interventions, which means that we need to have the capacity to deal with diseases in such a way as to enable greater and more egalitarian distribution of evidence-based healthcare solutions. This was the start of Medicine based not only on logic but also on logic associated with evidence.

Without doubt, Brazil is at the forefront of the process of developing and disseminating the culture of Evidence-Based Medicine. This culture has now been adopted by the World Health Organization and the healthcare systems of the United States, Canada, the United Kingdom and the countries of the European Union. Nonetheless, even though our country is following the vanguard of Evidence-Based Medicine, this does not mean that all the work has been done. Perhaps 25% of the pathway has been covered so far and there is still much to be done.
